# Novel Vegetation Indices to Identify Broccoli Plants Infected With *Xanthomonas campestris* pv. *campestris*

**DOI:** 10.3389/fpls.2022.790268

**Published:** 2022-06-23

**Authors:** Mónica Pineda, María Luisa Pérez-Bueno, Matilde Barón

**Affiliations:** ^1^Department of Biochemistry and Molecular and Cell Biology of Plants, Estación Experimental del Zaidín, Spanish National Research Council (CSIC), Granada, Spain; ^2^Department of Plant Physiology, Facultad de Farmacia, University of Granada, Granada, Spain

**Keywords:** biotic stress, climate change, hyperspectral reflectance imaging, machine learning, thermography

## Abstract

A rapid diagnosis of black rot in brassicas, a devastating disease caused by *Xanthomonas campestris* pv. *campestris* (Xcc), would be desirable to avoid significant crop yield losses. The main aim of this work was to develop a method of detection of Xcc infection on broccoli leaves. Such method is based on the use of imaging sensors that capture information about the optical properties of leaves and provide data that can be implemented on machine learning algorithms capable of learning patterns. Based on this knowledge, the algorithms are able to classify plants into categories (healthy and infected). To ensure the robustness of the detection method upon future alterations in climate conditions, the response of broccoli plants to Xcc infection was analyzed under a range of growing environments, taking current climate conditions as reference. Two projections for years 2081–2100 were selected, according to the Assessment Report of Intergovernmental Panel on Climate Change. Thus, the response of broccoli plants to Xcc infection and climate conditions has been monitored using leaf temperature and five conventional vegetation indices (VIs) derived from hyperspectral reflectance. In addition, three novel VIs, named diseased broccoli indices (DBI_1_-DBI_3_), were defined based on the spectral reflectance signature of broccoli leaves upon Xcc infection. Finally, the nine parameters were implemented on several classifying algorithms. The detection method offering the best performance of classification was a multilayer perceptron-based artificial neural network. This model identified infected plants with accuracies of 88.1, 76.9, and 83.3%, depending on the growing conditions. In this model, the three Vis described in this work proved to be very informative parameters for the disease detection. To our best knowledge, this is the first time that future climate conditions have been taken into account to develop a robust detection model using classifying algorithms.

## Introduction

The cultivation of broccoli (*Brassica oleracea* var. *italica*) has become increasingly attractive and profitable. It is highly regarded for its nutritional value and also its antioxidant and anticancer properties (Owis, 2015). In Spain, the production of broccoli has increased exponentially in the last decades, and it is expected to continue rising in future. In 2018, broccoli crop yields reached up to 561,000 tons in Spain, and most of them were exported to European countries (latest available data reported by Ministerio de Agricultura, Pesca y Alimentación, www.mapa.gob.es).

Pests and plant diseases are a great challenge in modern agriculture and the main cause of production and economic losses in agriculture worldwide (Savary et al., 2012). Current practices and social activities, such as intensified monoculture in large areas, the use of genetically uniform plant varieties, and international trading of agricultural commodities, contribute largely to the widespread of plant disease epidemics and rapid pathogen evolution (Zhan et al., 2015). Like other Brassica crops, broccoli plants are susceptible to infection by fungi (Alternaria leaf spot, anthracnose, blackleg, or mildews), some viruses (virus mosaic), and bacteria (black rot, soft rots, bacterial leaf spots). Among the bacterial pathogens, *Xanthomonas campestris* is one of the most important in brassicas (Mansfield et al., 2012; Ekman et al., 2014; Dep. Primary Industries and Regional Development, Government Western Australia 2018, www.agric.wa.gov.au/broccoli/diseases-vegetable-brassicas). The most notable pathovar of *X. campestris* is *campestris* (Xcc), which is the causal agent of black rot of crucifers and affects all cultivated brassicas. Indeed, and according to the report elaborated by EIP-AGRI Focus Group for the European Commission, Xcc is a threat to the production of broccoli, cauliflower, and cabbage throughout Europe (2016, Integrated Pest Management for Brassica, https://ec.europa.eu/eip/agriculture/en/publications/eip-agri-focus-group-ipm-brassica-final-report). Moreover, Xcc can be subdivided into nine races on the basis of the responses they induce on different cultivars. Among the nine races described for Xcc, races 1 and 4 are considered the most virulent and spread worldwide (Fargier and Manceau, 2007; Tortosa et al., 2018).

Precision agriculture demands the development of imaging sensor-based methods of detection and diagnosis of plant stress, including diseases. Several optical sensors are currently implemented to monitor crop fields (Aasen et al., 2019; Gerhards et al., 2019; Maes and Steppe, 2019; Pérez-Bueno et al., 2019a; Kashyap and Kumar, 2021; Pineda et al., 2021). Their applicability at lab scale and in high-throughput platforms by proximal sensing, and in the field by remote sensing, makes them particularly useful. However, the data provided by imaging sensors are large and complex and, consequently, difficult to interpret. Hence, improving our ability to extract useful information from these vast datasets requires the use of machine learning algorithms (Sperschneider, 2020). Machine learning is a subset of artificial intelligence (AI), consisting of algorithms that are able to learn patterns from a database of known samples and, based on those patterns, identify or categorize new samples. In agriculture, these algorithms can assist in the monitoring and decision-making processes of crop management (Chlingaryan et al., 2018; Golhani et al., 2018; Liakos et al., 2018; Gao et al., 2020). Thus, the implementation of imaging sensors and AI is a pivotal tool for crop management based on digital agriculture (Talaviya et al., 2020; Jung et al., 2021). However, alteration in growth conditions due to climate change imposes an additional challenge to plant disease detection methods based on AI. The expected rises in CO_2_ concentration and temperature associated to climate change would have an impact on agriculture, affecting plants and pathogen physiology (Trivedi et al., 2022) and their geographical distribution (Aidoo et al., 2021). For that reason, potential future climate should be considered as an experimental variable to develop more robust detection methods.

Thermography and multi/hyperspectral reflectance imaging are the most common sensors applied in agriculture (Zarco-Tejada et al., 2018; Maes and Steppe, 2019; Pérez-Bueno et al., 2019b). On the one hand, canopy to air differential temperature (T_C_-T_A_) is an indirect measurement of the vegetation transpiration rate (Scarth et al., 1948; Milthorpe and Spencer, 1957; Fuchs and Tanner, 1966) and is widely used in proximal and remote sensing for stress detection, as recently reviewed by Pineda et al. (2021). On the other hand, the high spectral resolution of hyperspectral reflectance imaging allows the creation of a growing collection of vegetation indices (VIs). These VIs are transformations of two or more spectral bands which allow reliable temporal and spatial inter-comparisons of vegetation attributes. Thus, VIs are quite simple and effective parameters to quantitatively and qualitatively evaluate vegetation traits such as vigor, fitness, and pigment composition, among other applications (Huete et al., 2002).

In fact, many VIs can be found in the literature. One of the most widely used is the normalized difference vegetation index (NDVI), which is related to vitality of canopies (Tucker, 1979; Pettorelli, 2013). Other VIs correlate with a wide range of plant physiological traits. For example, the photochemical reflectance index (PRI) correlates with photosynthetic activity (Gamon et al., 1992); the carotenoid reflectance index (CRI) (Gitelson et al., 2002) and the anthocyanin reflectance index (ARI) (Gitelson et al., 2001) are related to pigment contents; and the water balance index (WBI) is connected to water content in leaves (Peñuelas et al., 1993). Indeed, recent works have implemented VIs to the study of plants infected by *Xanthomonas* spp. Abdulridha et al. (2019) used a collection of VIs (ARI and NDVI among them) implemented on classifying algorithms to successfully identify tangerine plants infected by *X. citri* pv. *citri*. Similarly, NDVI values correlated well with the extension of the lesions caused by *X. campestris* pv. *oryzae* on rice leaves (Zhang et al., 2022). Moreover, several works have compared the association between climate change and the interannual variability registered on NDVI in several locations around the world (Kalisa et al., 2019; Bagherzadeh et al., 2020; Zhao et al., 2021). Nonetheless, new reflectance parameters could be defined for a given purpose to maximize differences when standard VIs are not sensitive enough (Miao et al., 2007; Mahlein et al., 2013; Zhang et al., 2017; El-Hendawy et al., 2019; Jia et al., 2019; Yuan et al., 2019).

The main aim of this work was to develop an efficient method for the detection of Xcc infection in broccoli plants based on thermal and hyperspectral reflectance imaging on individual leaves. For this purpose, six parameters were recorded: leaf temperature (particularly T_C_-T_A_) and five already known VIs. Moreover, three novel VIs specifically designed for detecting the Xcc infection were extracted from the reflectance spectra of healthy and diseased broccoli leaves. They were named diseased broccoli indices (DBI_1_-DBI_3_). This set of nine parameters were implemented on a selection of algorithms widely used on precision agriculture for their success in classifying infected plants: the multilayer perceptron-based artificial neural network (MLP), the support vector machine (SVM), and the k-nearest neighbor (kNN). Finally, the suitability of the trained models was evaluated by comparing their performances in correctly classifying new samples as healthy or diseased leaves under a range of climate conditions, including intermediate and extreme climate change scenarios, as well as current climate conditions. Furthermore, the relevance of every input parameter for the detection of Xcc infection in broccoli plants was evaluated.

## Materials and Methods

### Plant Growth at Different Climate Conditions

Growth conditions under two possible future climate change scenarios were chosen taking into account the 5th Assessment Report by the Intergovernmental Panel on Climate Change (AR5; IPCC, 2014). In that assessment, a range of projections of greenhouse gases emissions responding to both socio-economic development and climate policy was considered. Future climate conditions were estimated based on representative concentration pathways (RCPs), depending on potential scenarios of greenhouse gases emissions and their atmospheric concentrations, air pollutant emissions, and land use for the year 2100. Thus, current climate conditions (CCC) were compared to future scenarios, being RCP 8.5 the most extreme scenario, meaning most dramatic increase in CO_2_ levels and subsequent global warming. In turn, the so called RCP 4.5 would represent an intermediate scenario between CCC and RCP 8.5 and was considered by the AR5 as the most probable scenario by 2100.

The C_3_ broccoli plants (*Brassica oleracea* var. *italic* cv. *calabrese natalino*) were grown in a growth chamber in a 16/8-h day/night regime with 60% relative humidity, 200 mol photon m^−2^ s^−1^ of PAR light. The ambient temperature and CO_2_ concentrations (Table 1) were chosen according to the data regionalized by the Spanish State Meteorology Agency (AEMet) for Region of Murcia (largest Spanish broccoli producer) for current climate conditions and those corresponding to RCP 4.5 and RCP 8.5 in years 2081–2100. Day and night temperatures correspond to the average values in Region of Murcia during the growing season. For each experiment, plants were sown and grown at the corresponding CCC, RCP 4.5, or RCP 8.5 conditions.

**Table 1:** Climatic conditions assessed for broccoli growth: CCC (current climate conditions), RCP 4.5, and RCP 8.5 (Representative Concentration Pathways 4.5 and 8.5) regionalized for Region of Murcia for years 2081–2100.

**Climate Scenario**	**Temperature (** **°** **C)**		**CO_**2**_ (ppm)**
	**Day**	**Night**	
CCC	31	17	408
RCP 4.5	34	20	650
RCP 8.5	37	23	1000

### Bacterial Growth and Inoculation

*Xanthomonas campestris* pv *campestris* (Xcc) race 1 and race 4 were grown for 24 h at 28°C in LB (Luria-Bertani) plates. Bacterial suspensions were prepared in sterile 10 mM MgCl_2_ at 10^8^ colony-forming units per ml (cfu mL^−1^) by adjusting the optical density at 600 nm to 0.1.

**Figure 1:**
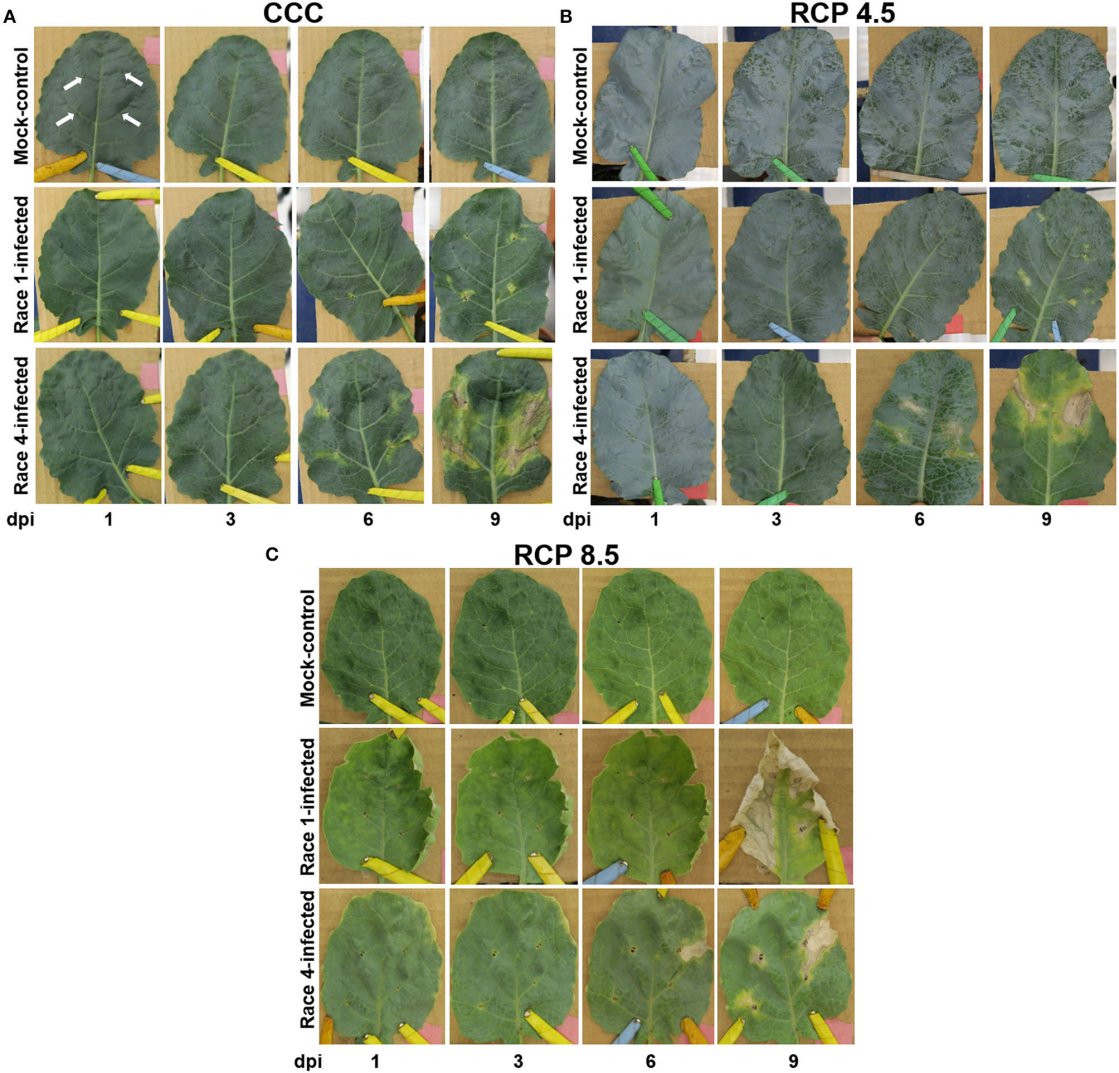
Timecourse of symptoms evolution of mock-control, Xcc race 1-infected, and Xcc race 4-infected broccoli plants at current climate conditions (**A**; CCC), and the representative concentration pathways RCP 4.5 **(B)** and RCP 8.5 **(C)**. White arrows indicate the inoculation points. For simplicity, they have only been shown in mock-controls leaves at CCC. Dpi, days post-inoculation.

The third leaf of four-week-old plants (under CCC or RCP 4.5) or five-week-old plants (in case of RCP 8.5) was mock-inoculated with sterile 10 mM MgCl_2_ or inoculated with bacterial suspension by clipping four secondary veins per leaf with rat tooth tweezers previously dipped in the corresponding solution (Figure 1). For each experiment, twelve plants per treatment (CCC and RCP 4.5) and four plants per treatment (RCP 8.5) were inoculated. Leaves were imaged at 1, 2, 3, 6, and 9 days post-inoculation (dpi). At least two experiments per climate condition were carried out, providing similar results.

### Thermal Imaging

Thermal images of whole leaves were recorded using a FLIR A305sc camera (FLIR Systems, Wilsonville, OR, USA) vertically positioned 30 cm above the leaf, according to Pérez-Bueno et al. (2016). For each measurement, 10 thermal images were collected in the plant growth chamber over 10 s. These images were averaged to extract temperature values for whole leaves. Image processing was carried out using the FLIR ResearchIR v. 3.4 software.

### Hyperspectral Reflectance Imaging

Reflectance spectra (400–1,000 nm) of broccoli leaves were recorded using a Pika L hyperspectral imaging camera (Resonon, Bozeman, MT, USA) in the visible (400–700 nm) to near-infrared spectral range (700–1,000 nm), with a spectral sampling at 2.1 nm and a spectral resolution (full width at half maximum) of 3.7 nm. The camera was positioned vertically 45 cm over the sample, which was placed on a translation stage. Thus, a datacube with 281 images was built for each attached leaf.

Leaves were illuminated with four calibrated xenon lamps with homogeneous light intensity between 400 and 1,000 nm, positioned above the samples and around the camera. Previous to leaf measurements, dark and light corrections were made in darkness and illuminating a white homogenous calibration tile provided by Resonon, respectively. Dark and light corrections, build-up of datacubes and analysis, were carried out with the software Spectronon v. 2.134 (Resonon).

Reflectance spectra averaged for whole leaves were obtained and used to calculate images corresponding to several widely used VIs, as summarized in Table 2. To avoid overfitting of machine learning models, only not redundant VIs were selected.

**Table 2:** Common vegetation indices (VIs) from the literature that were used in this work.

**VIs name**	**Related to**	**Equation**	**References**
Anthocyanins reflectance index 1	Anthocyanins	$\text{ARI}=\frac{1}{\text{R}550}\;-\;\frac{1}{\text{R}700}$	Gitelson et al., 2001
Carotenoids reflectance index 2	Carotenoids	$\text{CRI}=\frac{1}{\text{R}510}\;-\;\frac{1}{\text{R}700}$	Gitelson et al., 2002
Normalized difference vegetation index	Vigor	$\text{NDVI }=\;\frac{\text{R}800\;-\text{ R}670}{\text{R}800\;+\text{ R}670}$	Tucker, 1979
Photochemical reflectance index	Photosynthesis	$\text{PRI }=\;\frac{\text{R}531\;-\text{ R}570}{\text{R}531\;+\text{ R}570}$	Gamon et al., 1992
Water balance index	Water	WBI = R900−R970	Peñuelas et al., 1993

### Data Analysis

Numerical data obtained from thermal and reflectance images (including reflectance spectra and VIs) were managed using Microsoft Office Excel 2016 (Microsoft Corporation, Redmond, WA, USA). Aiming to design a simple method of detection, values were averaged from whole leaves rather than regions of interest.

Two-tailed Student's *t*-test (Microsoft Excel) was performed to compare, for every treatment and at every dpi assayed: (a) spectra reflectance profiles; (b) values of novel VIs (DBIs). The null hypothesis was that there were no differences between treatments. This hypothesis was considered false at *p* < 0.05, and variables were treated as different when p-value was below this value. Figure graphs were plotted using Microsoft Excel.

### Classification Analysis by Machine Learning

Data collected through whole experiments were organized in databases (Microsoft Excel), one per climate condition. Each database contained values of selected parameters (T_C_-T_A_, NDVI, PRI, ARI, CRI, WBI, DBI_1_, DBI_2_, and DBI_3_) at every dpi (1–9 dpi) and treatment (mock-control, Xcc race 1-, and Xcc race 4-infected plants). In addition, data were rescaled from zero to one to ensure comparison between treatments and days, according to the equation: *rescaled value* = *(x-minimum)/maximum*. Then, the three databases were randomly partitioned into training and test datasets, in a proportion of 7:3, respectively (Table 3). This partition was carried out using a seed that ensured that every category (treatment and dpi) was well represented in both datasets. The experimental data were analyzed by the free version of KNIME v. 4.3.2 (KNIME AG, Zurich, Switzerland; www.knime.com; Berthold et al., 2008).

**Table 3:** Sample size (n) of the training and test databases created for each growth condition.

**Treatment**	**Training dataset (*n*)**	**Test dataset (*n*)**
CCC	98	41
RCP 4.5	84	39
RCP 8.5	42	18

Three models were built for each one of the three growing conditions by analyzing the corresponding databases with three supervised classifying algorithms: MLP, SVM, and kNN. MLP is a network inspired by biological neural networks in which different interconnected nodes (called neurons) organized in layers transmit information to each other, learning from both input and output data (Hahn, 2009; Behmann et al., 2015). In contrast, SVM distributes samples in a high-dimensional feature space defined by support vectors. In this case, new samples are categorized based on what side of hyperplanes they fall on (Behmann et al., 2015). Finally, kNNs assign proportional weights to the contributions of the sample neighbors based on distances. These weights determine to what category a new sample would most likely belong to (Blanzieri and Melgani, 2008).

Broccoli leaves were categorized into mock-control, Xcc race 1-, and Xcc race 4-infected plants using the classifying algorithms MLP, SVM, and kNN. The performance of classification was evaluated in terms of (i) sensitivity (true positive rate); (ii) specificity (true negative rate); (iii) accuracy (percentage of right guesses); and (iv) *F*-measure (harmonic mean of precision and sensitivity; where precision is the number of correct control samples divided by the number of all plants classified as “control”). All the three feedforward backpropagation MLPs tested were designed to have one hidden layer with four neurons (half the number of variables used to feed them). Higher number of hidden layers or their neurons did not result in an improvement of the performance. A polynomial kernel was used for the SVMs, with bias = 1 and gamma = 1. More complex spatial kernels did not improve the performance of the algorithm. Finally, the optimal number of neighbors for the kNN algorithm was *k* = 5 using the Euclidean distance. Regarding SVM and kNN libraries, we have used the basic nodes (SVM learner and K nearest neighbor, respectively) implemented on Knime software v. 4.3.2. This process was performed independently for each of the three climate conditions under study.

Finally, the global variable importance was calculated for each parameter (T_C_-T_A_, NDVI, PRI, ARI, CRI, WBI, DBI_1_, DBI_2_, and DBI_3_), that is, how informative was a given parameter for the model to make a correct decision. For such a purpose, global surrogate random forest (RF) models were trained to estimate the variable importance using the Global Feature Importance component developed for Knime software (https://hub.knime.com/knime/spaces/Examples/latest/00_Components/Model%20Interpretability/Global%20Feature%20Importance~xsR90ymhRbHOc78Z). RF was trained on the standardly pre-processed input data. Feature importance was then calculated by counting how many times it had been selected for a split and at which rank (level) among all available features (candidates) in the trees of the RF.

## Results

### Evolution of Symptoms Under a Range of Growth Conditions

Plants grown from sowing under CCC, RCP4.5, or RCP8.5 conditions were inoculated with Xcc race 1 or 4, and the evolution of symptoms was followed up to 9 dpi. At CCC and RCP 4.5, Xcc infection on broccoli plants consisted in chlorosis followed by a progressive necrosis of the tissue surrounding the inoculation site to finally reach the V-shaped lesions typical of this bacterial infection (Figure 1). Xcc race 4 caused the most severe symptoms, with necrosis at the clipping point starting at 3 dpi and evident at 6 dpi; chlorosis surrounded the inoculated area at 6 dpi, and V-shaped lesions were patent at 9 dpi. In contrast, Xcc race 1 produced similar symptoms in a slower time course, with a delay of 3 days. Mock-control leaves only displayed the actual lesions. It is worth noticing that there was no evolution in symptoms from 0 to 1 dpi under any of the assayed growth conditions.

The RCP 8.5 conditions affected the growth of broccoli plants, which displayed stunting and early senescence. Moreover, leaves were smaller and thicker than those of plants grown at CCC or RCP 4.5 conditions. At RCP 8.5, the evolution of the infection by either race resembled that described for Xcc race 4 under CCC. Moreover, Xcc race 1 was more virulent than Xcc race 4 at 9 dpi.

### Novel VIs Could Discriminate Between Healthy and Xcc-Infected Broccoli Leaves

**Figure 2:**
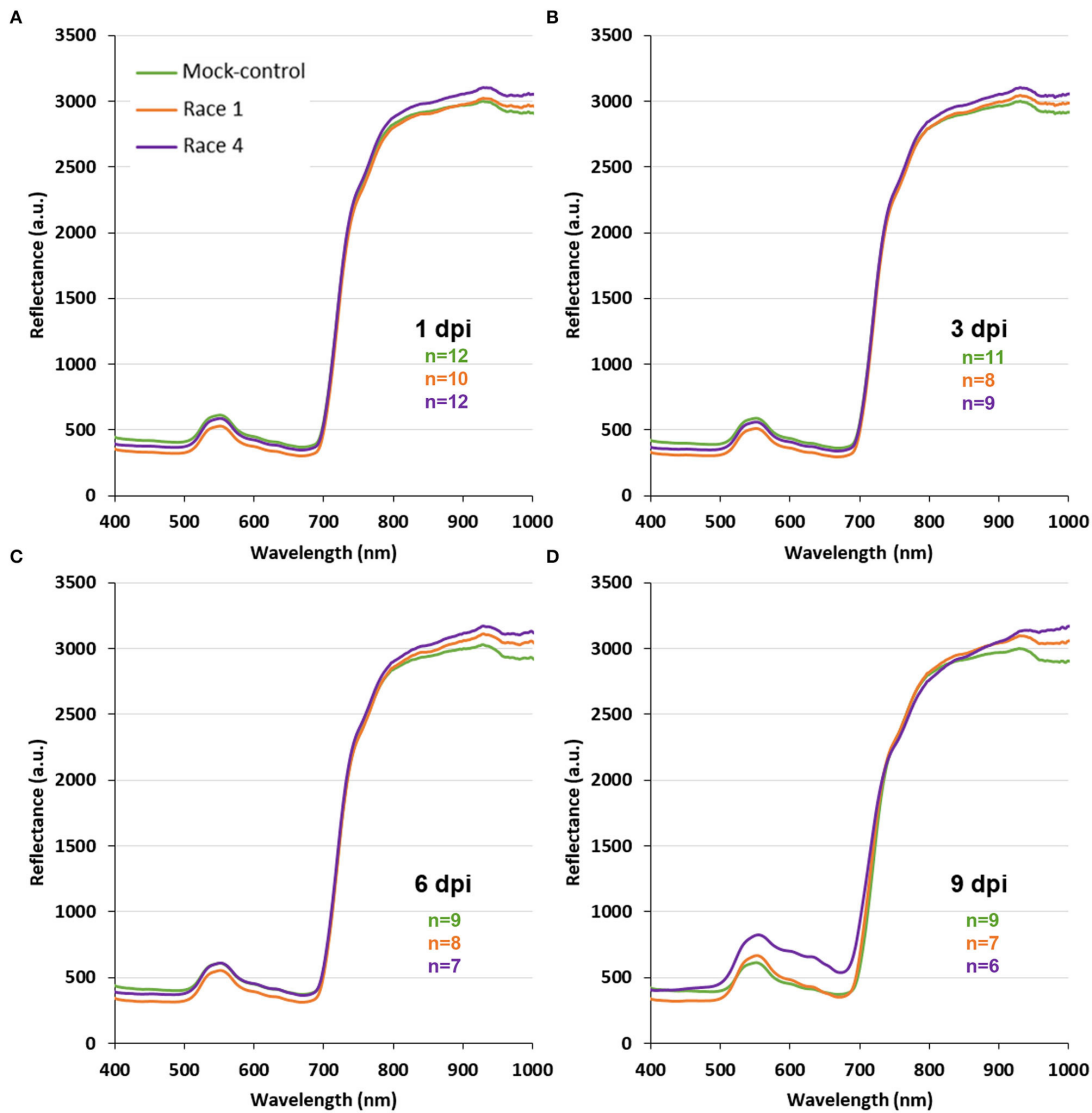
Spectral profiles of whole leaves of mock-control, Xcc race 1-infected, and Xcc race 4-infected broccoli plants at current climate conditions and at different days post-inoculation (dpi). Graphs represent mean values for every treatment. Sample size (*n*) is given for each timepoint and treatment: mock-control (green); Xcc race 1-infected (orange); and Xcc race 4-infected broccoli plants (purple). **A, B, C**, and **D**: 1, 3, 6, and 9 dpi, respectively.

For every treatment (mock-control, Xcc race 1-, and Xcc race 4-infected), whole leaf reflectance spectra were registered at 1, 2, 3, 6, and 9 dpi. Those profiles revealed specific spectral patterns for each treatment, showing clear differences between them from the first timepoint measured (Figure 2; Supplementary Figures 1, 2). Thus, the use of VIs based on hyperspectral reflectance measurements seemed to be useful to distinguish between control and Xcc (race 1 or 4)-infected plants. The common VIs ARI, CRI, PRI, NDVI, and WBI were obtained from the spectra for every treatment and dpi measured and recorded in a database for each climate condition.

In addition, novel VIs were designed, based on spectral differences between healthy and Xcc-infected broccoli leaves. Reflectance spectra were compared by Student's t-test in order to determine at which wavelengths reflectance values were statistically different (at least *p* < 0.05) between treatments. Thus, three spectral ranges were found to show maximal differences between treatments at every dpi tested: 400–500, 600–700, and 900–1000 nm. On the contrary, the regions 520–570 and 730–890 nm were very stable and not affected by the infection until 9 dpi. For this reason, they were selected for “normalization” of the designed parameters. Several wavelengths were chosen within these spectral regions of interest. To avoid redundancy of adjacent wavelengths in the reflectance spectra, only wavelengths separated by at least 40 nm were used in this process. Finally, the selected wavelengths were combined by different mathematical calculations (additions, subtractions, divisions, or combinations thereof) in order to find novel VIs showing statistical differences (*p* < 0.05 according to Student's *t*-test) between treatments along entire experiments at each climate condition. Among the large collection of proposed VIs, three of them, named diseased broccoli indices 1–3 (DBI_1−3_; Table 4), offered maximum significant differences between mock-control and Xcc-infected broccoli plants. It is worth noticing that DBI_1−3_ did not show statistically significant correlation with each other, meaning that DBI_1−3_ did not provide redundant information to the classifiers.

**Table 4:** Novel vegetation indices (VIs) firstly described in this work.

**VIs name**	**Related to**	**Equation**
Diseased broccoli index 1	Xcc infection	${\text{DBI}}_{1}\;=\;\frac{\text{R}400\;-\text{ R}690}{\text{R}850}$
Diseased broccoli index 2	Xcc infection	${\text{DBI}}_{2}\;=\;\frac{\text{R}400}{\text{R}850}$
Diseased broccoli index 3	Xcc infection	${\text{DBI}}_{3}\;=\;\frac{\text{R}578}{\text{R}529}$

### Identification of Xcc-Infected Leaves by Classificatory Algorithms

For each experimental condition, an independent database was built containing the values of selected parameters (T_C_-T_A_, NDVI, PRI, ARI, CRI, WBI, DBI_1_, DBI_2_, and DBI_3_) for every treatment and dpi assayed. The three databases were normalized and then randomly split in two datasets: training (70%) and testing (30%). Each of the three training dataset was used to feed supervised classifying algorithms (MLP, SVM, and kNN) to classify samples into the following categories: mock-control, Xcc race 1-, and Xcc race 4-infected leaves; each of the three testing dataset was used to calculate their performance of classification.

**Figure 3:**
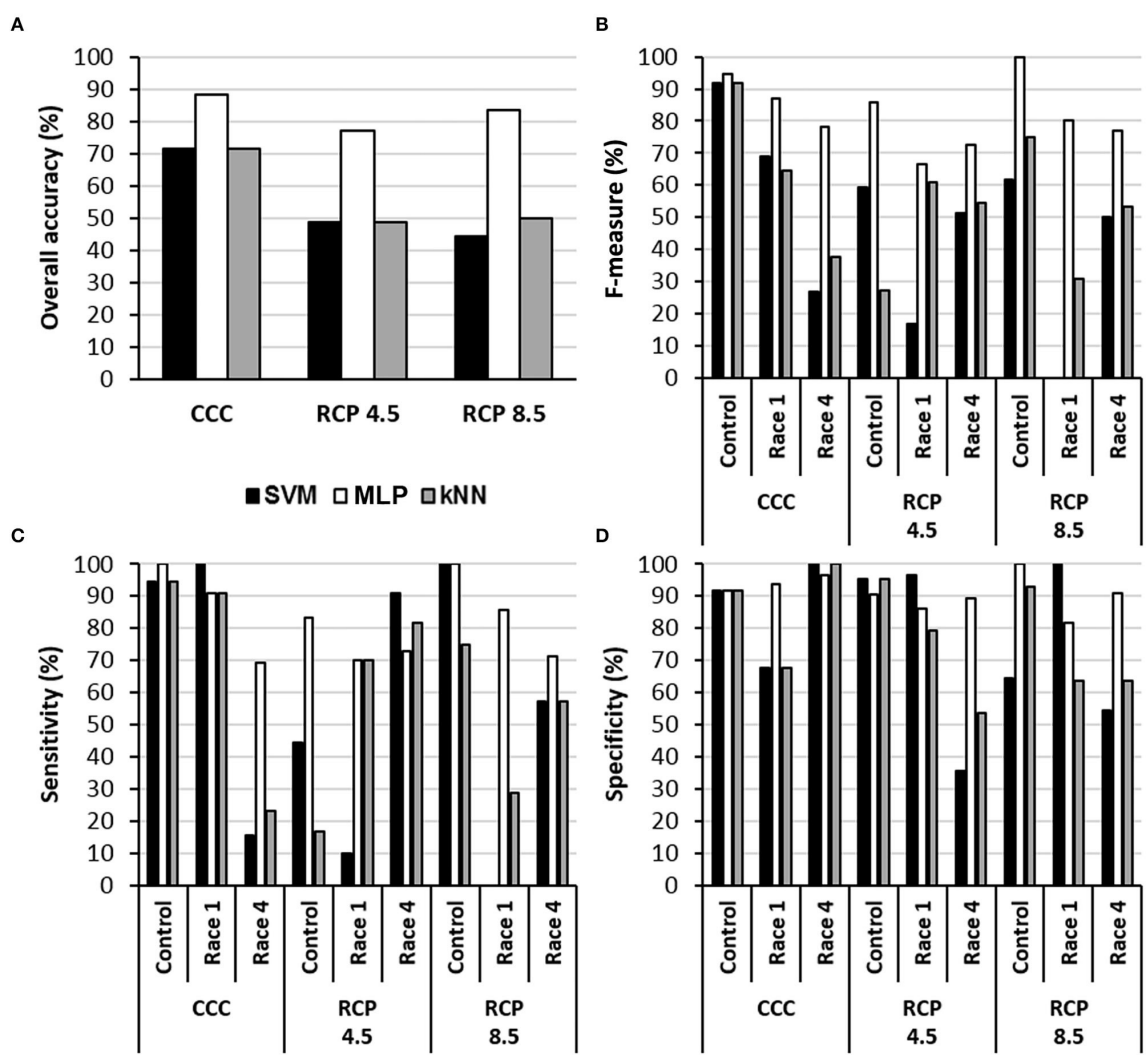
Performance of three algorithms for the classification of samples into the categories control, Xcc race 1-infected, and Xcc race 4-infected leaves in terms of overall accuracy **(A)**, *F*-measure **(B)**, sensitivity **(C)**, and specificity **(D)** for each climate scenario. SVM, support vector machine; MLP, multilayer perceptron-based artificial neural network; kNN, k-nearest neighbors (*k* = 5 neighbors). CCC, current climate conditions; RCP 4.5 and 8.5, representative concentration pathways 4.5 and 8.5.

The MLPs provided the highest accuracy for every climate condition (Figure 3). They also provided the highest *F*-measure under every condition and, in general terms, the highest sensitivity. In contrast, SVM and kNN showed similar accuracies for CCC; however, these two algorithms were not able to identify control and infected samples at RCP 4.5 or RCP 8.5, with accuracies of 45–50% and rather low specificity. Moreover, any attempt of classification by MLP, SVM, or kNN into two categories (mock-control vs Xcc-infected) was inefficient. This was probably due to the underrepresentation of mock-control samples in the datasets (1/3 healthy vs. 2/3 of infected), whereas in three-category models, every group had the same size.

**Figure 4:**
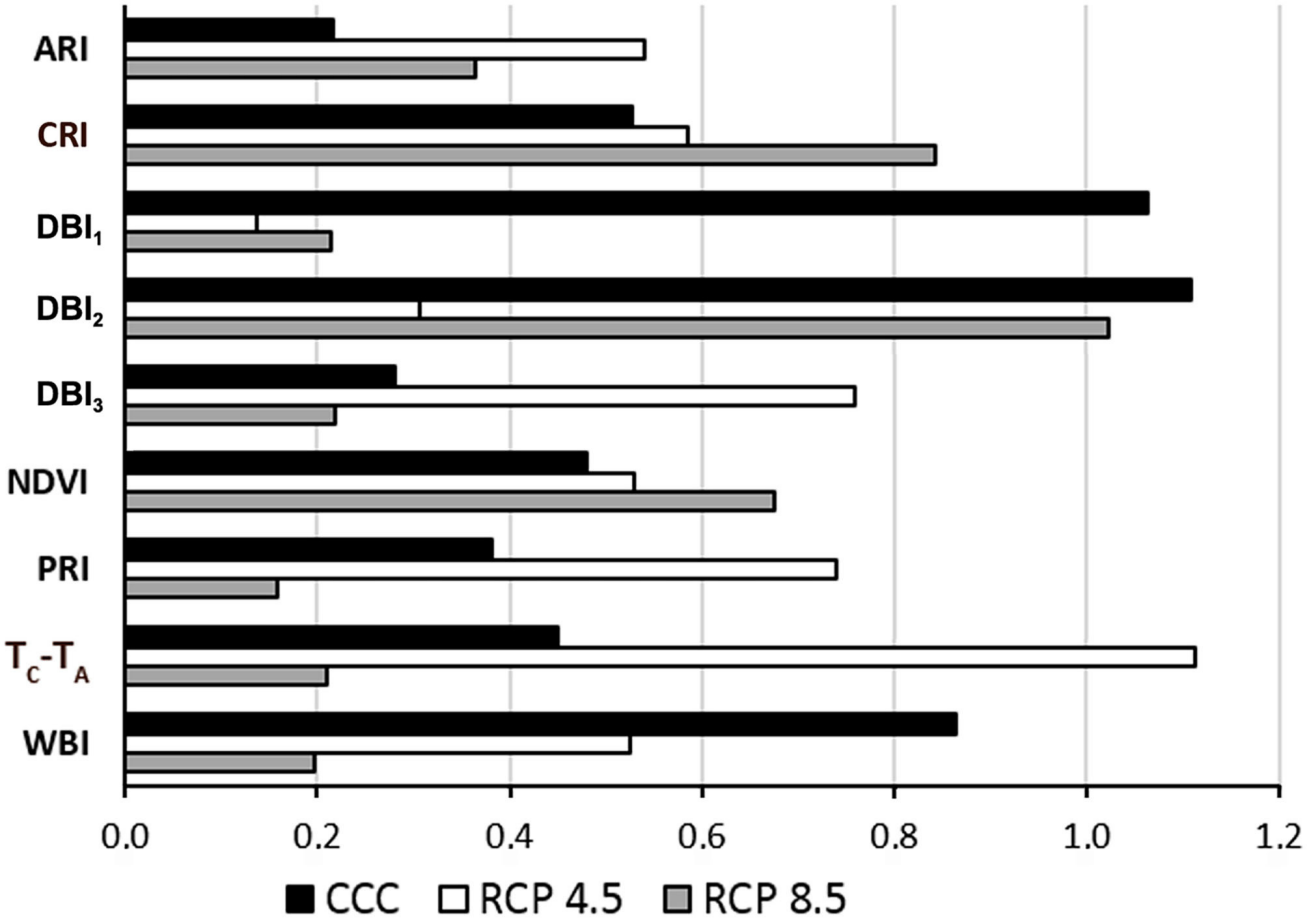
Global variable importance calculated using a global surrogate random forest (RF) model. CCC, current climate conditions; RCP 4.5 and 8.5, representative concentration pathways 4.5 and 8.5; ARI, anthocyanin reflectance index; CRI, carotenoid reflectance index; DBI_1−3_, disease broccoli index 1–3; NDVI, normalized difference vegetation index; PRI, photochemical reflectance index; T_C_-T_A_, normalized temperature; WBI, water balance index.

The suitability of the designed VIs for the identification of Xcc-infected leaves was evaluated in terms of global variable importance in the classifiers, calculated by a surrogate RF algorithm (Figure 4). The accuracies obtained for the fit of each RF were 85.7%, 87.2%, and 88.9% for CCC, RCP 4.5, and RCP 8.5, respectively. Under CCC, the most informative parameters were DBI_1_, DBI_2_, and WBI. In contrast, under RCP 4.5 conditions, T_C_-T_A_, DBI_3_, and PRI obtained the highest global variable importance. Finally, under RCP 8.5 conditions, DBI_2_, CRI, and NDVI were the most instructive parameters.

## Discussion

Imaging techniques appear to be essential for precision agriculture due to their fast time-spatial response to biotic and abiotic stress in a non-destructive manner (Barón et al., 2016; Mahlein, 2016). In the last years, thermal and (multi- or hyper-) reflectance imaging sensors have been broadly used for monitoring stress in crop fields. Furthermore, sustainable agriculture is increasingly relying on AI (such as classifying algorithms) coupled with computer vision, to solve farming issues and to promote the automation of decision-making process (Tian et al., 2020; Nabwire et al., 2021). However, these methods require basic research to define informative parameters that efficiently report the health state and fitness of a particular crop. This work aims to define optimal VIs and classifiers for the detection of Xcc-infected broccoli leaves. Furthermore, the robustness of those models was analyzed under climate conditions mimicking those expected for Region of Murcia in years 2081–2100.

According to Fargier and Manceau (2007), when a cultivar of a brassica is infected by Xcc, a collection of polymorphisms is obtained depending on the race inoculated. In the case of broccoli plants used for this study (*B. oleracea* var. *italic* cv. *calabrese natalino*), the symptoms developed under CCC triggered by races 1 and 4 were similar. However, Xcc race 1 showed a slower timecourse than race 4 under the same ambient conditions. The process of undergoing climate change could affect considerably plant biochemistry and therefore plant defense responses. For this reason, it is relevant to include future climate conditions in disease detection studies. Indeed, each disease may respond differently to these variations, and thus, climate change would cause neutral, positive, or negative effects on plant responses to diseases (Trebicki et al., 2017; Velásquez et al., 2018; Cheng et al., 2019). According to the results reported here, symptoms caused by Xcc on susceptible broccoli plants would not be altered on an intermediate climate change scenario like RCP 4.5. These results are in accordance with previous works, as extensively reviewed by Gullino et al. (2018). However, the RCP 8.5 imposed a stress condition limiting or slowing down plant growth. It will be of particular interest to gain knowledge about the impact of combined high temperature and high CO_2_ on photosynthetic processes of the broccoli plant which, as a C_3_ species, is well adapted to mild temperatures. Nevertheless, further research would be needed to fully understand the impact of climate change on broccoli plant physiology (particularly on photosynthesis), as well as on the physiology of Xcc races, and/or their interaction with host plants.

In literature, a number of classic VIs derived from multispectral (and hyperspectral) imaging can be found. This VIs can be used to detect, classify, and quantify specific diseases with varying degrees of success (Lowe et al., 2017). However, high-resolution spectra recorded with hyperspectral imaging sensors allow the selection of an optimized set of wavelengths to maximize differences between healthy and infected plants. Those wavelengths can be used to create novel parameters specific for a given host–pathogen system or stress factor. This approach has already been demonstrated to be suitable to detect diseased plants when combined with AI algorithms. Thus, Mahlein et al. (2013) reported specific spectral disease indices for the detection of sugar beet plants infected with Cercospora leaf spot, sugar beet rust, or powdery mildew. Those indices improved disease detection and identification when implemented on classifiers. Similarly, Yuan et al. (2019) proposed a novel method for detecting anthracnose in tea plants based on hyperspectral imaging that included two new disease indices in the classificatory models. Moreover, the analysis of reflectance spectral data of healthy and diseased wheat ears allowed the creation of a novel index that demonstrated a stronger ability to determine the severity of the Fusarium head blight compared with other sixteen existing spectral indices (Zhang et al., 2020).

In this work, three novel VIs have been developed to successfully distinguish between healthy and Xcc-infected broccoli plants (Table 4). Leaf reflectance is a complex phenomenon dependent on biochemical and biophysical properties of the canopy leaves, which in turn are affected by growth conditions and diseases. Thus, the visible reflectance range (400–700 nm) is mostly influenced by the leaf pigment content; the reflectance in the near-infrared range (700–1100 nm) depends on water content and leaf structure, or internal scattering processes; and the short-wave infrared (1,100–2,500 nm) is influenced by the composition of leaf chemicals and water, as reviewed by Mahlein (2016). Since chlorophylls are the main pigments influencing reflectance spectrum at 400 and 690 nm, both DBI_1_ and DBI_2_ indices could be indicative of the severity of chlorosis. Moreover, DBI_3_ could be also related to the contents on chlorophylls and carotenes (Carter and Knapp, 2001).

DBI_1_, DBI_2_, and DBI_3_, together with thermal (T_C_-T_A_) and other common hyperspectral reflectance parameters (NDVI, PRI, ARI, CRI, and WBI), were implemented in three different supervised classifiers (MLP, SVM, and kNN) for each experimental condition. Since the learning process of each algorithm differs from each other, so will the quality of its predictions on the new samples. In this sense, it is a common procedure to compare the performance of several algorithms when sorting new samples (the validation datasets) after training on the same dataset. Metrics such as specificity (true negative rate), sensitivity (true positive rate), accuracy (percentage of right guesses), or *F*-measure (harmonic mean of precision and sensitivity) evaluate the performance of classification, that is, the estimation of the true risk of error of the output prediction of a machine learning algorithm (Shalev-Shwartz and Ben-David, 2014; Liakos et al., 2018). The MLPs were the most effective classifier, with the highest overall accuracy and *F*-measure under the three growing conditions assayed. The performance of the MLP models was comparable to that reported by other authors for disease detection classifiers. Indeed, an increasing number of studies apply classifiers to spectral data (including or not thermal parameters) to identify infected plants at conditions resembling CCC. For example, Sankaran et al. (2013) reported an accuracy of 87% when classifying citrus trees infected by *Candidatus Liberibacter* spp, a bacteria causing Huanglongbing disease. Zarco-Tejada et al. (2018) obtained accuracies of disease detection exceeding 80% when classifying *Xylella fastidiosa*-infected olive trees. This pathogen, alike Xcc, is a xylem bacterium. Abdulridha et al. (2020a,b) identified tomato plants infected with tomato yellow leaf curl virus, *Xanthomonas perforans*, or *Corynespora cassiicola* (a fungus) with 94–100% accuracy depending on the pathogen. Pérez-Bueno et al. (2019b) detected avocado trees suffering white root rot (caused by the fungus *Rosellinia necatrix*) with accuracies up to 82.5%. Nguyen et al. (2021) achieved accuracies ranging from 82 to 96.75% when identifying vines affected by the Grapevine vein-clearing virus. Similarly to the results here reported, Yuan et al. (2019) designed novel hyperspectral reflectance indices which help to identify *Gloeosporium theae-sinesis* Miyake-infected tea plants with 94 and 98% accuracies at pixel and leaf levels, respectively.

The performance of the models was affected differentially by growing conditions, depending on the classifier. In both RCPs, the accuracy of the classifiers decreased in all cases. However, the accuracy of models based on MLP only decreased from 88.1% at CCC to 76.9 and 83.3% for RCP 4.5 and RCP 8.5, respectively. This advantage of MLPs against SVM and kNN models could be related to the fact that MLPs are less affected by noise factors (compared to other algorithms), which in turn reduces significantly the influence of the unknown variability. Therefore, MLPs are usually more robust models that often outperform other classifying algorithms in solving a variety of classification problems (Basheer and Hajmeer, 2000; Bala and Kumar, 2017). To our best knowledge, this is the first time that machine learning classifiers have been applied to hyperspectral and thermal data taken under climate conditions mimicking those projected for the future in order to classify healthy and infected plants.

DBI_1_, DBI_2_, and DBI_3_ proved to be important features for plant classification according to a surrogate RF used for testing the global importance of variables. Overall, DBI_1_ and DBI_2_ were the most informative parameters of the set for CCC. However, the global variable importance varied for each parameter depending on the climate conditions. Under RCP 4.5 conditions, T_C_-T_A_ and DBI_3_ were the most informative parameters. In contrast, DBI_2_ was the parameter with the highest global variable importance at RCP 8.5. This effect may be due to the impact of growing conditions on the symptomatology (degree of chlorosis and/or necrosis) of the infections, as discussed above.

## Conclusion

The parameters DBI_1_, DBI_2_, and DBI_3_ here presented are good reporters for Xcc infection in broccoli leaves. Furthermore, the model based on MLP and the set of parameters DBI_1_, DBI_2_, and DBI_3_ along with common VIs (ARI, CRI, NDVI, PRI, and WBI) and T_C_-T_A_ would be an effective procedure for the identification of Xcc-broccoli infected plants. In addition, this model proved to be robust regardless of the climate conditions.

## Data Availability Statement

The original contributions presented in the study are included in the article/Supplementary Material. Further inquiries can be directed to the corresponding authors.

## Author Contributions

MP, MLP-B, and MB conceived and designed the experiments and wrote the manuscript. MP and MP-B conducted the experiments. MP analyzed the data, interpreted the results, and mounted images. MB contributed to the materials, equipment, and analysis tools. All the authors reviewed it and approved the final version.

## Funding

This work was supported by grant RTI2018-094652-B-I00 funded by MCIN/AEI/10.13039/501100011033 and by “ERDF: A way of making Europe”. The free open access publication was partially funded by Consejo Superior de Investigaciones Científicas (CSIC) through the Unidad de Recursos de Información Científica para la Investigación (URICI).

## Conflict of Interest

The authors declare that the research was conducted in the absence of any commercial or financial relationships that could be construed as a potential conflict of interest.

## Publisher's Note

All claims expressed in this article are solely those of the authors and do not necessarily represent those of their affiliated organizations, or those of the publisher, the editors and the reviewers. Any product that may be evaluated in this article, or claim that may be made by its manufacturer, is not guaranteed or endorsed by the publisher.

## Supplementary Material

Supplementary Figure 1 Spectral profiles of whole leaves of mock-control, Xcc race 1-infected, and Xcc race 4-infected broccoli plants at RCP 4.5 and at different days post-inoculation (dpi). Graphs represent mean values for every treatment. Sample size (*n*) is given for each timepoint and treatment: mock-control (green); Xcc race 1-infected (orange); and Xcc race 4-infected broccoli plants (purple).

Supplementary Figure 2 Spectral profiles of whole leaves of mock-control, Xcc race 1-infected, and Xcc race 4-infected broccoli plants at RCP 8.5 and at different days post-inoculation (dpi). Graphs represent mean values (*n* = 4) for every treatment and timepoint.
