# Supplementary figures and images for: Novel Vegetation Indices to Identify Broccoli Plants Infected With Xanthomonas campestris pv. campestris
Source: Front Plant Sci. 2022 Jun 23;13:790268. doi: 10.3389/fpls.2022.790268 (PMC9265216; doi:10.3389/fpls.2022.790268)

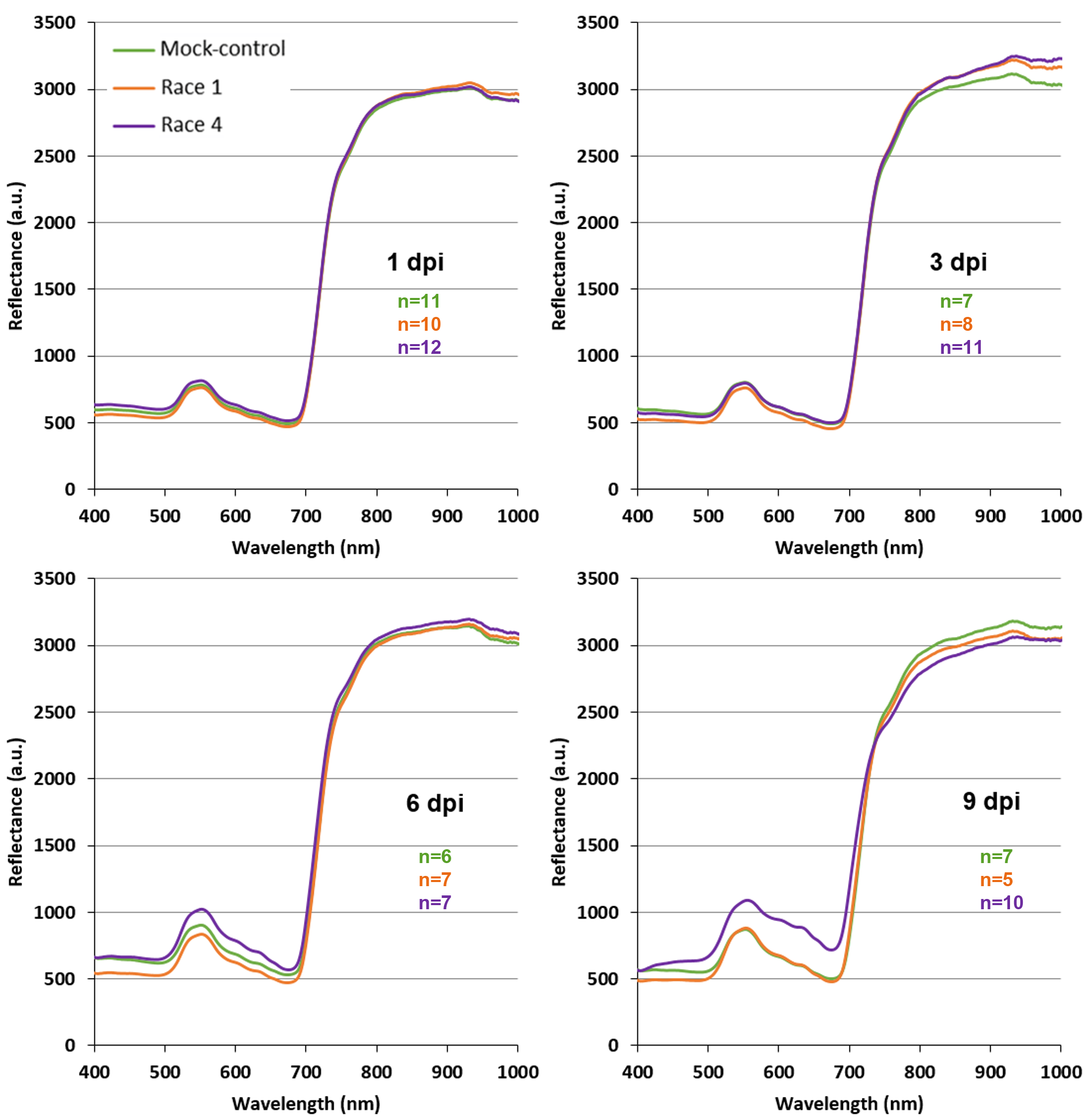

Supplement: Supplementary Figure 1 — Spectral profiles of whole leaves of mock-control, Xcc race 1-infected, and Xcc race 4-infected broccoli plants at RCP 4.5 and at different days post-inoculation (dpi). Graphs represent mean values for every treatment. Sample size (n) is given for each timepoint and treatment: mock-control (green); Xcc race 1-infected (orange); and Xcc race 4-infected broccoli plants (purple). [file Image_1.TIF]

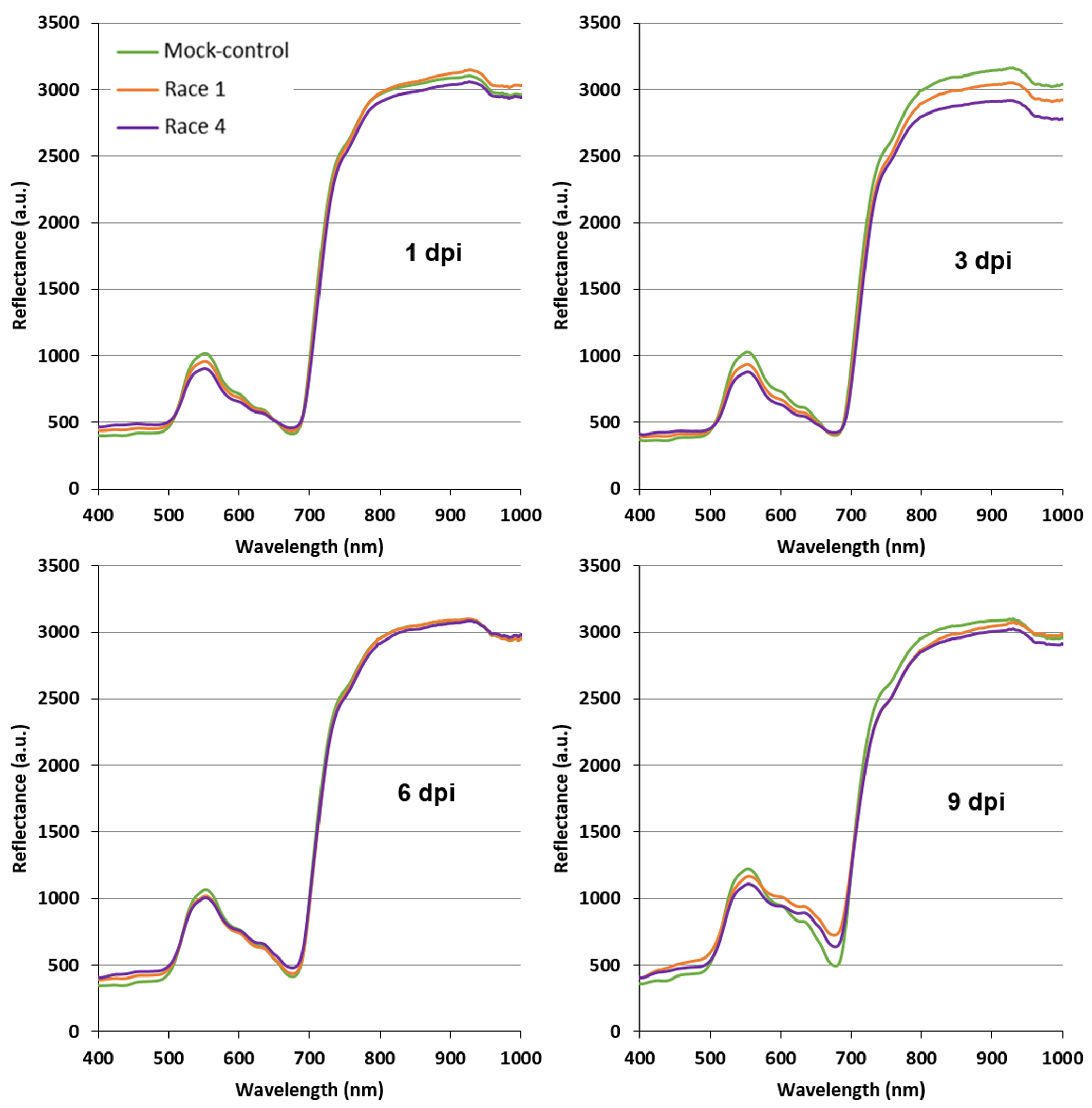

Supplement: Supplementary Figure 2 — Spectral profiles of whole leaves of mock-control, Xcc race 1-infected, and Xcc race 4-infected broccoli plants at RCP 8.5 and at different days post-inoculation (dpi). Graphs represent mean values (n = 4) for every treatment and timepoint. [file Image_2.TIF]
